# A prognostic signature based on seven T-cell-related cell clustering genes in bladder urothelial carcinoma

**DOI:** 10.1515/med-2023-0773

**Published:** 2023-09-18

**Authors:** Jie Yang, Fenghai Zhou, Xia Yang, Pengcheng Ma, Xiaoling Ma

**Affiliations:** The First School of Clinical Medicine, Lanzhou University/Department of Reproductive Medicine, The First Hospital of Lanzhou University, Lanzhou, Gansu, 730000, P.R. China; The First School of Clinical Medicine, Lanzhou University/Department of Urology, Gansu Provincial Hospital, No. 204 Donggang West Road, Chengguan District, Lanzhou, Gansu, 730000, P.R. China; Department of Reproductive Medicine, The First Hospital of Lanzhou University, Lanzhou, Gansu, 730000, P.R. China

**Keywords:** bladder urothelial carcinoma, immune microenvironment, prognosis, single-cell RNA sequencing

## Abstract

Bladder urothelial carcinoma (BLCA) is one of the most common cancer-related deaths in the world, along with high mortality. Due to the difficult detection of early symptoms, the treatment for this disease is still dissatisfactory. Thus, the current research hotspot is beginning to focus on the immune microenvironment in this disease, aiming to provide guidance for diagnosis and treatment. In this study, the single-cell RNA sequencing data downloaded from the gene expression omnibus database was used to classify the immune cells of BLCA. And the final seven T-cell-related cell clustering genes associated with BLCA prognosis (*HSPA2*, *A2M*, *JUN*, *PDGFRB*, *GBP2*, *LGALS1*, and *GAS6*) were screened out, and then used for constructing the prognostic model against BLCA based on the Cox and LASSO regression analysis. Satisfactorily, the model could efficiently evaluate the overall survival of BLCA and had the potential to be applied for the clinic treatment. Moreover, we also revealed that the difference in immune infiltration levels and gene mutation might account for the diverse prognosis in BLCA patients. In a word, our findings provided a novel insight for designing efficient immunotherapies for BLCA.

## 1 Introduction

Bladder urothelial carcinoma (BLCA) is a common urinary system malignancy, along with a higher incidence and mortality [1,2]. It has been reported that 61,700 new cases were diagnosed in men and even 12,120 deaths occurred in 2022 in the United States [3]. More seriously, approximately 550,000 new cases, as well as 200,000 deaths, were happened each year [4]. Though the advances involved in the clinical therapies including surgery and chemo-radiotherapy have been developed, the poorer overall survival of 5-year in BLCA was also unsatisfactory [4,5]. Recently, more and more attention has been focused on this disease, hoping to reveal novel diagnostic methods and therapeutic targets.

The tumor immune microenvironment (TME) is always flooded with immunosuppressive cells and inhibitory cytokines, leading to effective immune cells being unable to infiltrate and recognize the tumor and even lose the ability to fight cancer [6]. Previous studies identified a series of immune-related key genes involved in the occurrence, development, and prognosis of human cancer including BLCA. For example, Li et al. found that CD248 was highly and specifically expressed in tumor-associated vessels in BLCA and demonstrated that the level of CD248 contributed to predict the BLCA prognosis [7]. Zhu et al. revealed that *MTHFD2* was an oncogene in BLCA, and its expression level was closely correlated with the poor prognosis of patients and severe immune infiltrates [8]. In addition, several prognostic models were constructed based on different immune genes including inflammatory response-associated genes [9] and ferroptosis-related genes [10]. Although these prognostic models were demonstrated that could efficiently predict the overall survival of BLCA patients, more different models were also needed and these could be crossly validated in order to increase accuracy.

Here, we downloaded the single-cell RNA sequencing data of BLCA from the gene expression omnibus (GEO) database and screened out the differentially expressed genes in T cells. Then, a prognosis model based on the key genes was constructed. According to our results, we confirmed that the prognosis model could accurately predict the overall survival of BLCA patients and was potentially used for the clinical directed treatment. Meanwhile, the immune infiltration and gene mutation were analyzed between high- and low-risk groups, and we found that these different levels of immune infiltration and gene mutation probability might account for the diverse prognosis in BLCA patients. Our analysis might provide new insight for the clinical BLCA treatment.

## 2 Materials and methods

### 2.1 The data source

Transcriptome data, typed BLCA-FPKM, included 412 BLCA samples and clinical data from the cancer genome atlas (TCGA) database. We also downloaded GSE13507 data including BLCA samples and clinical data, and the single-cell sequencing dataset GSE145140 of BLCA from the GEO database.

### 2.2 Quality of single-cell sequencing

The condition was set as follows: The number of genes per cell ranged from 300 to 7,000, and the percentage of mitochondria-related genes was <10, the percentage of erythrocytes was <3, and the total gene expression copy number was less than 300,000.

### 2.3 Cluster analysis

Principal component analysis (PCA) was performed on the highly variable genes as previously described [11], the number of PCs was set to 6, and a total of 10 clusters were obtained. These clusters are presented in UMAP format, and the top 10 significantly different genes in each cluster are selected for mapping.

### 2.4 The construction of prognostic model

Univariate Cox proportional hazard regression analysis was performed to screen out key genes that were associated with BLCA prognosis in the last section, with *p* < 0.001 as the set. The prognostic model (Risk score) was constructed as follows:

\[\text{Risk}\hspace{.25em}\text{core}=\mathop{\sum }\limits_{i=1}^{n}\text{coefi}\times \text{id}]\]

Then patients were divided into high- and low-risk groups according to the median-risk score.

### 2.5 Evaluation of the prognosis model

Kaplan–Meier survival was used to analyze the overall survival of patients in two groups in the TCGA and the GEO datasets. The time ROC package was used to draw time-dependent ROC curves for 1 and 3 years. In addition, the heat maps were used to compare the expression of the model genes between two risk groups in two datasets. The distribution of patients between two risk groups was explored with the Rtsen package.

### 2.6 Evaluation of immune infiltration level between two risk groups

As previously described [12], the CIBERSORT web portal (http://CIBERSORT.stanford.edu/) was used to assess the immune cell subsets distribution, which was an algorithm that transformed the normalized expression matrix into infiltrating immune cell components [13].

### 2.7 Evaluation of gene mutation

MafTools package was used to evaluate the mutations in patients between two risk groups in two datasets as previously described [14].

### 2.8 Statistical analysis

R V4.1.0 (http://www.Rproject.org) was used to perform the statistical evaluation, with *p* < 0.05 as the significant threshold.

## 3 Results

### 3.1 The acquisition of important genes correlated to immune subtypes

**Figure 1:**
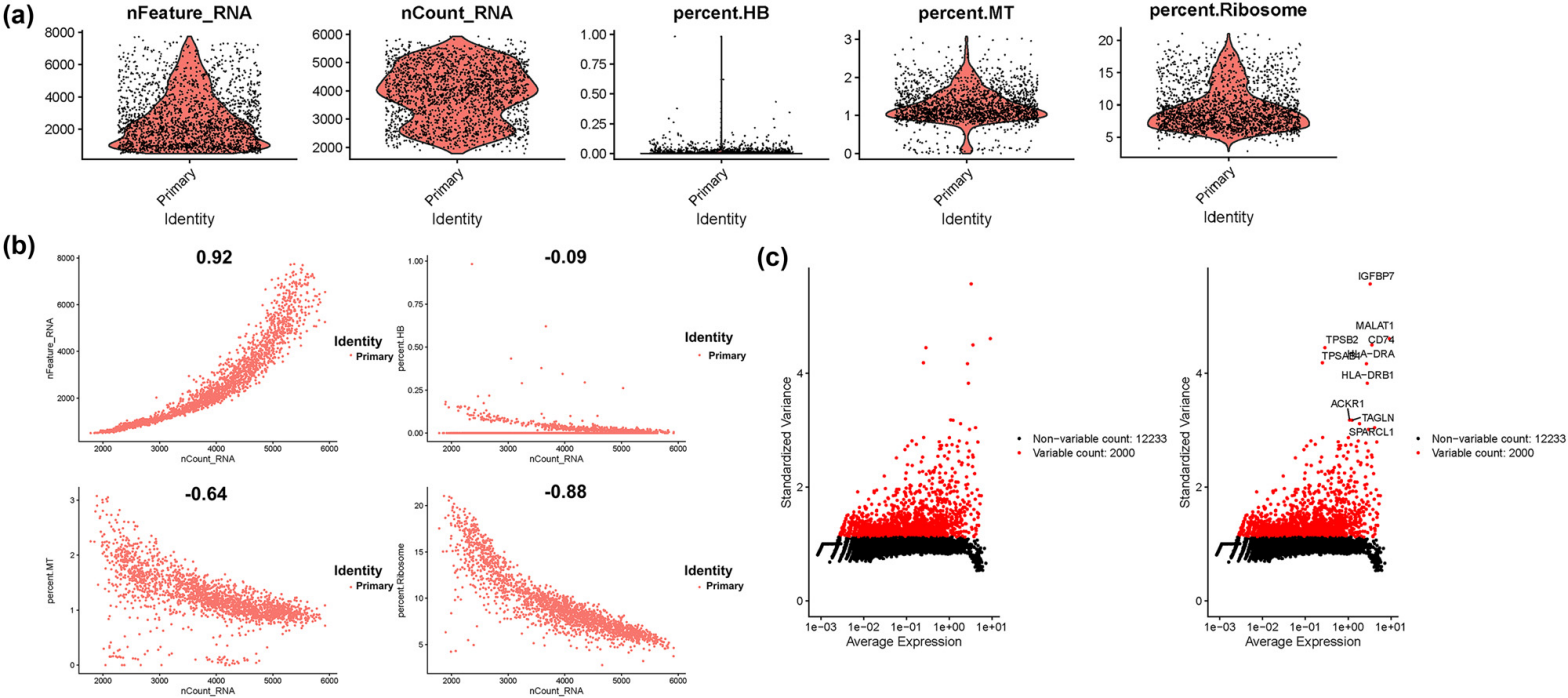
Quality control of single-cell RNA sequencing data. (a) Gene expression levels in each cell among four samples. (b) Scatter plot showing the distribution of count number (nCount_RNA) with gene number (nFeature_RNA), percentage of hemoglobin genes (percent.HB), percentage of mitochondrial genes (percent.MT), and percentage of ribosome genes (percent.Ribosome). (c) Scatter plots showing the 2000 high variable genes in red, and the top 10 genes were flagged.

First, we performed quality control on single-cell RNA sequencing data, and the number of genes expressed in all cells obtained by quality control will be 300–7,000. The distribution was relatively uniform. Meanwhile, mitochondrial genes were expressed in less than 5% of the cells, and cells expressing less than 1% of the hemoglobin gene (Figure 1a). From Figure 1b, we found that cells are evenly distributed in the four samples. The gene number is negatively correlated with their percent. HB level (−0.09), percent. MT (−0.64), and percent. Ribosome (−0.88). Hence, we selected the 2,000 high variable genes in red in Figure 1c, with the first top 10 genes flagged.

**Figure 2:**
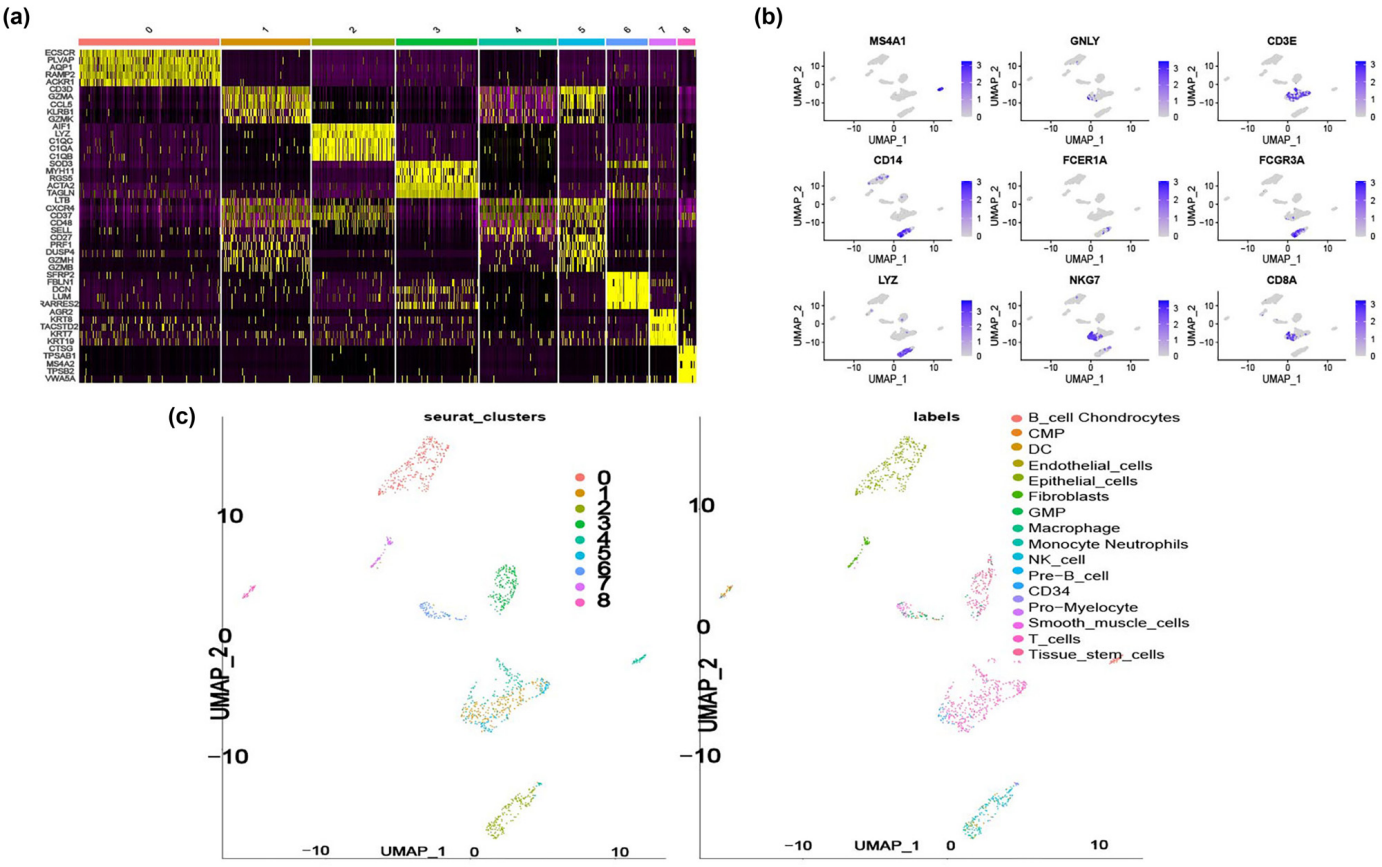
The acquisition of T-cell-related genes. (a) Heatmap showing the expression level of the top five marker genes of each cluster. (b) Umap plots showing the expression level of important marker genes in nine clusters. (c) Umap plots showing the cell-type identification of each cluster.

Then, through performing the PCA analysis, our analysis yielded the top ten genes with expression differences between the clusters (Figure 2a). The expression levels of the 10 genes were significantly higher than others in each cluster (Figure 2a). The expression of important biomarker genes is shown in Figure 2b. T cells or NK cells (markers: *MS4A1* and *CD3E*), B cells (*MS4A1*), and myeloid cells (*LYZ*) were clustered according to immune cell markers (*FCER1A* and *FCGR3A* are Macrophage markers; *GNLY* are Neutrophils marker; *CD8A* and *NKG7* are T-cell and NK-cell markers). The label colors according to separate clusters are shown in Figure 2c. Hence, T-cell-related genes were selected for the subsequent analysis.

### 3.2 Analysis of T-cell-related genes

**Figure 3:**
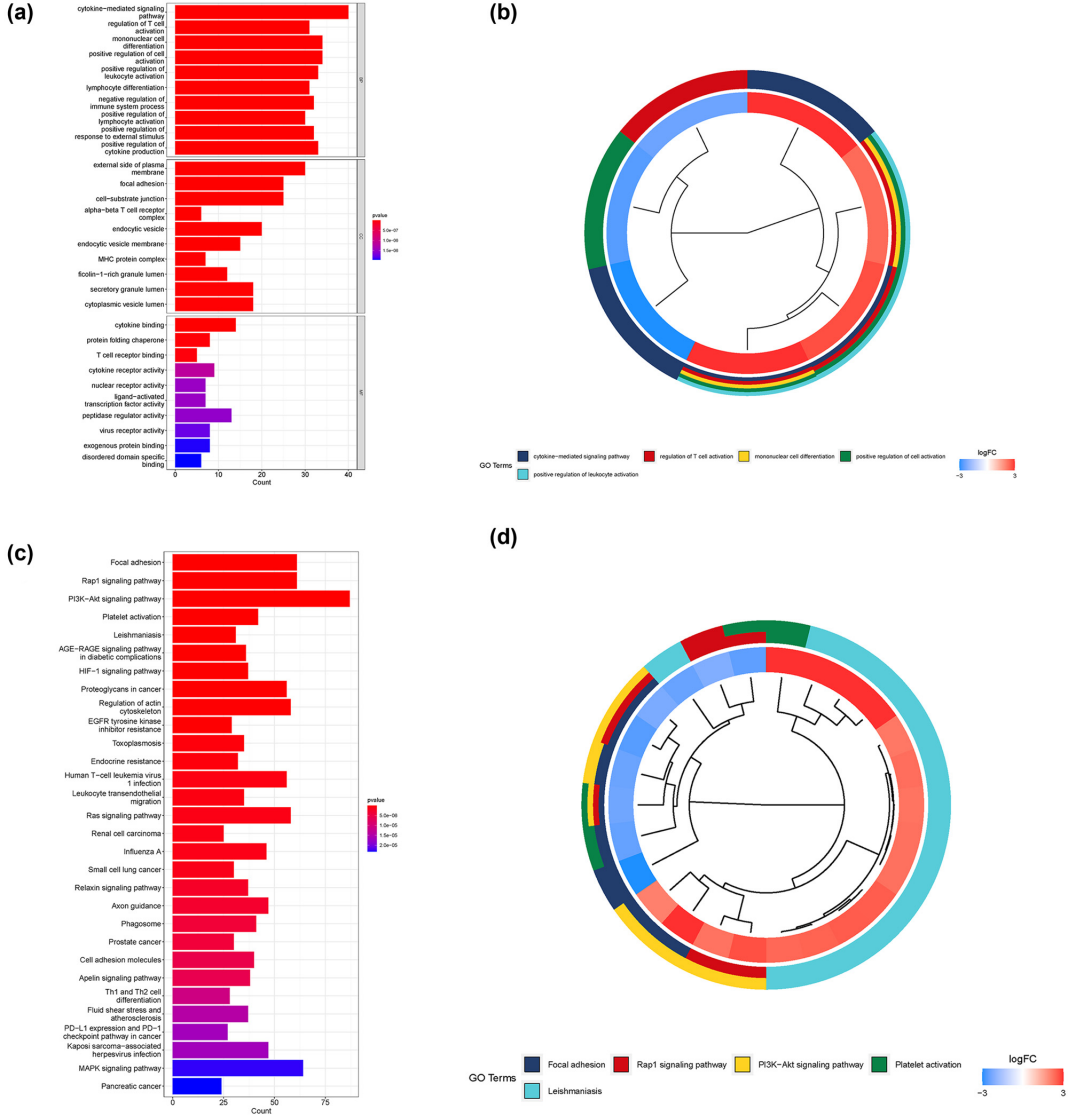
Enrichment analysis of T-cell-related genes. (a) Bar plot showing the GO analysis of T-cell-related genes. (b) Circos plot showing the log_2_ fold change of the main GO terms between BLCA samples and healthy samples. (c) Bar plot showing the KEGG enrichment analysis of T-cell-related genes. (d) Circos plot showing the log_2_ fold change of the main KEGG enrichment terms between BLCA samples and healthy samples.

To investigate which function and signaling pathways were enriched, KEGG and GO analyses were performed using these T-cell-related genes. As shown in Figure 3a and b for GO analysis, we found that these genes were significantly correlated to cytokine-mediated signaling pathway, regulation of T-cell activation, mononuclear cell differentiation, and positive regulation of cell activation. As shown in Figure 3c and d, these genes were significantly enriched in focal adhesion, Rap1 signaling pathway, and PI3K-Akt signaling pathway.

### 3.3 Construction of the prognostic model

**Figure 4:**
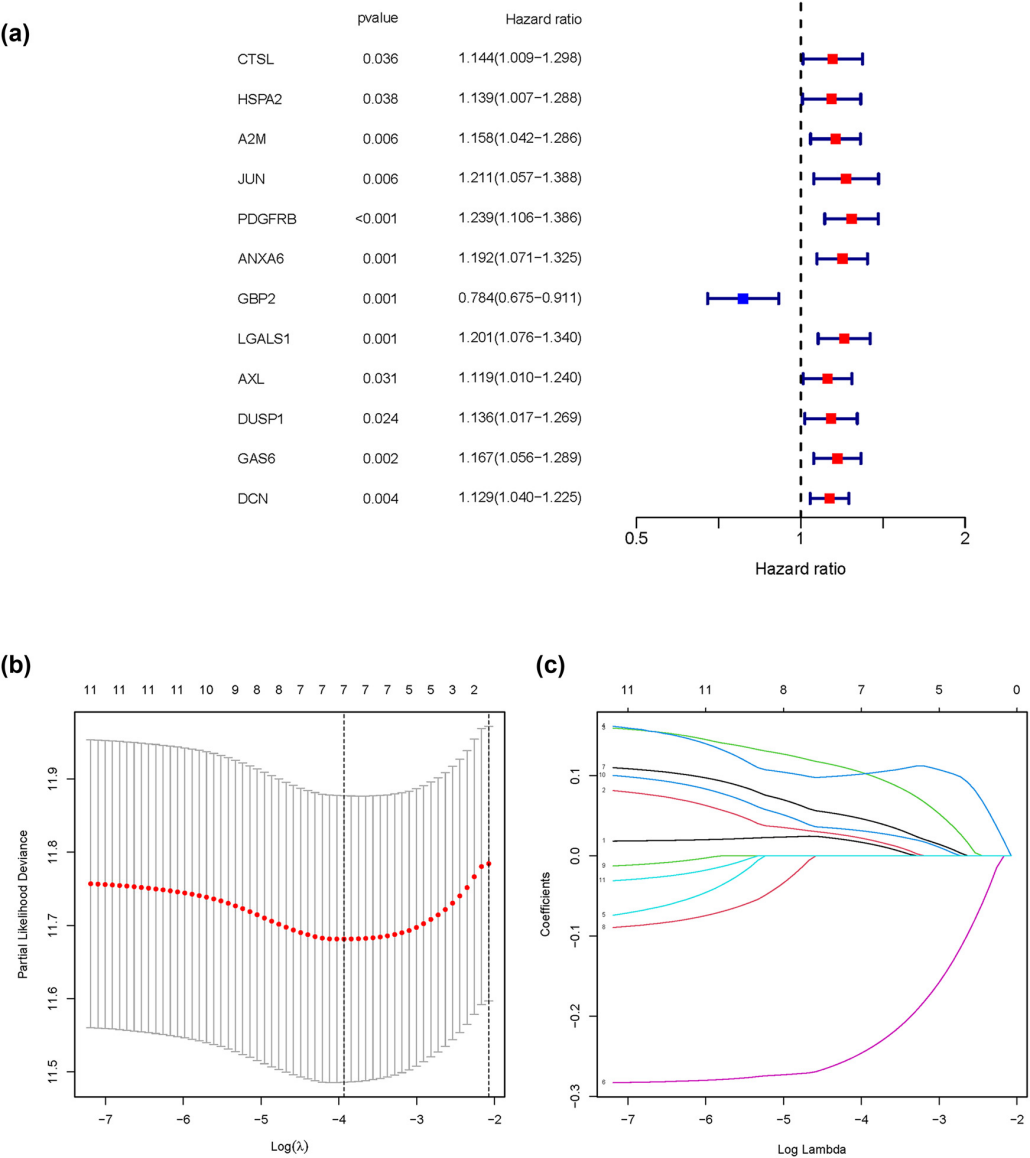
Construction of the prognostic model. (a) Scatter plot showing the univariate COX analysis for screening those genes related to T immune cells with prognostic values. (b) and (c) Line chart showing the LASSO regression analysis of the 12 genes.

Next, Cox analysis was done in conjunction with clinical follow-up data and identified 12 genes that were associated with T cells and correlated with survival time and status (Figure 4a). LASSO regression analysis was then operated based on these 12 genes (Figure 4b and c). We finally generated seven modeling genes, which were *HSPA2*, *A2M*, *JUN*, *PDGFRB*, *GBP2*, *LGALS1*, and *GAS6* for constructing the prognosis model, and then, the Risk score = *HSPA2 ×* 0.016 + *A2M ×* 0.020 + *JUN ×* 0.102 + *PDGFRB ×* 0.103 + *GBP2 ×* (−0.243) + *LGALS1 ×* 0.045 + *GAS6 ×* 0.030 was established. To better evaluate the value of our prognosis model, we divide the BLCA patients into high- and low-risk groups based on the median-risk score. *HSPA2* gene silencing could affect the motility and invasiveness of urothelial and cervical cancer cell lines (PMID: 19914824). Extracellular vesicle-derived *A2M* has been regarded as a novel diagnostic biomarker for bladder cancer (PMID: 36176383); here, we verified it as a target gene in the BLCA prognosis model. The oncogenic transcription factor *JUN* affects transcriptome regulation and cellular function, especially extracellular stimulation and energy metabolism in BLCA. The expression of T-cell-related *PDGFRB* was associated with tumor microenvironment, which significantly downregulated after chemotherapy treatment. *GBP2* is a member of the GBP family, which plays an essential role in the inflammatory process and might be a prognostic protective factor for BLCA (PMID: 17980030). Highly consistent with our single-cell data analysis, a previous study reported that bladder cancer cells with *LGALS1* silencing and *GAS6* depletion could reduce cell proliferation, lower invasive capability, and lower clonogenicity (PMID: 27440446 and PMID: 32547108).

### 3.4 The evaluation of the prognostic model

**Figure 5:**
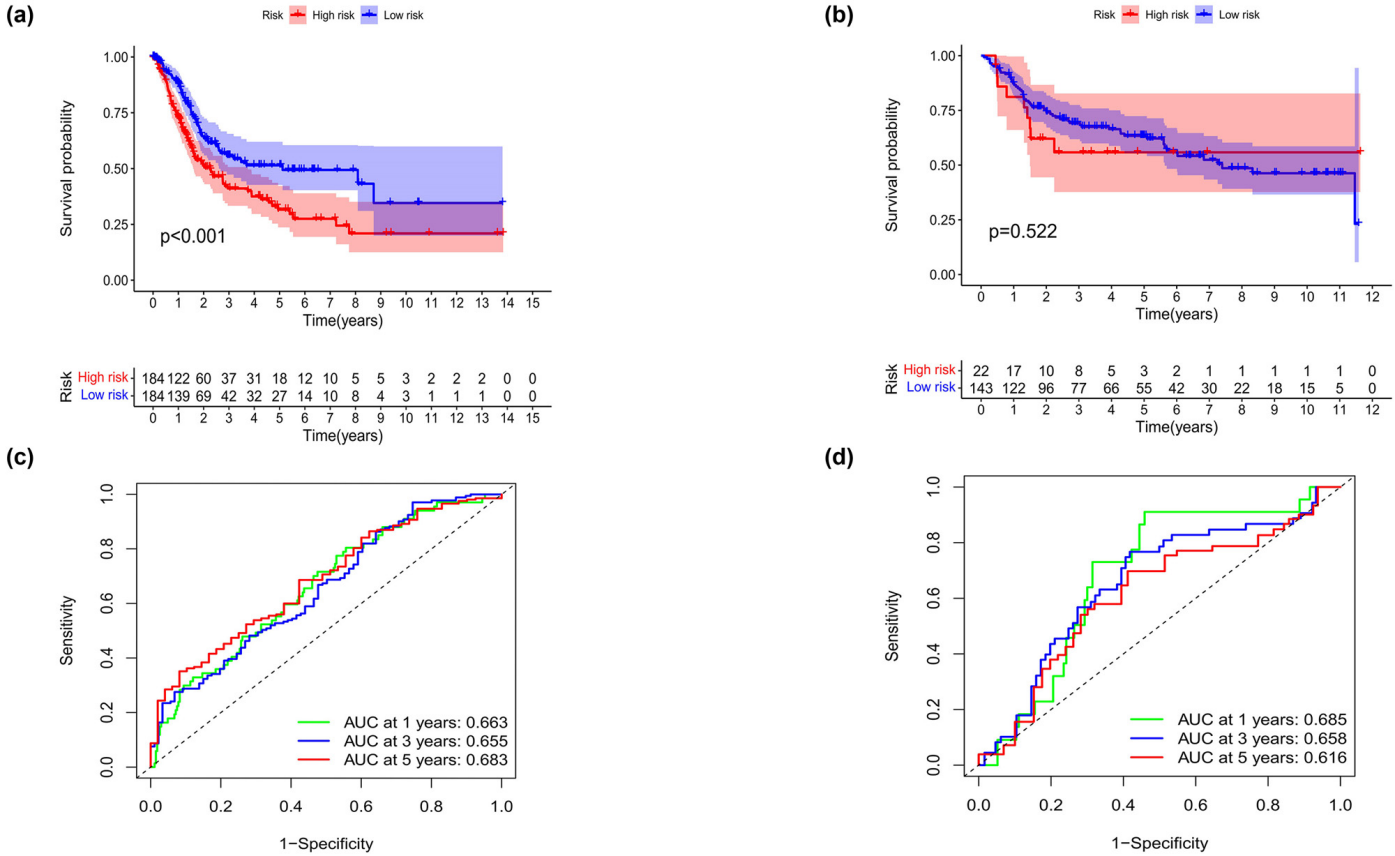
Evaluation of the value of the prognostic model. (a) and (b) The Kaplan–Meier survival curve analysis showing the significant difference between the high-risk and the low-risk groups in TCGA and GEO data sets. (c) and (d) Time-dependent ROC analysis showing the significant prognostic value in TCGA and GEO data sets.

Kaplan–Meier survival curves were plotted based on the database from TCGA and GEO datasets, and we found that the patients showed a poorer prognosis in the high-risk group both in the TCGA (Figure 5a) and in the GEO data sets (Figure 5b). The area under the ROC curve of 1 year and 3 years in both data sets was approximately 0.65, and that of 5 years was approximately 0.68 (Figure 5c and d), suggesting that the prognostic model has good stability and accuracy.

**Figure 6:**
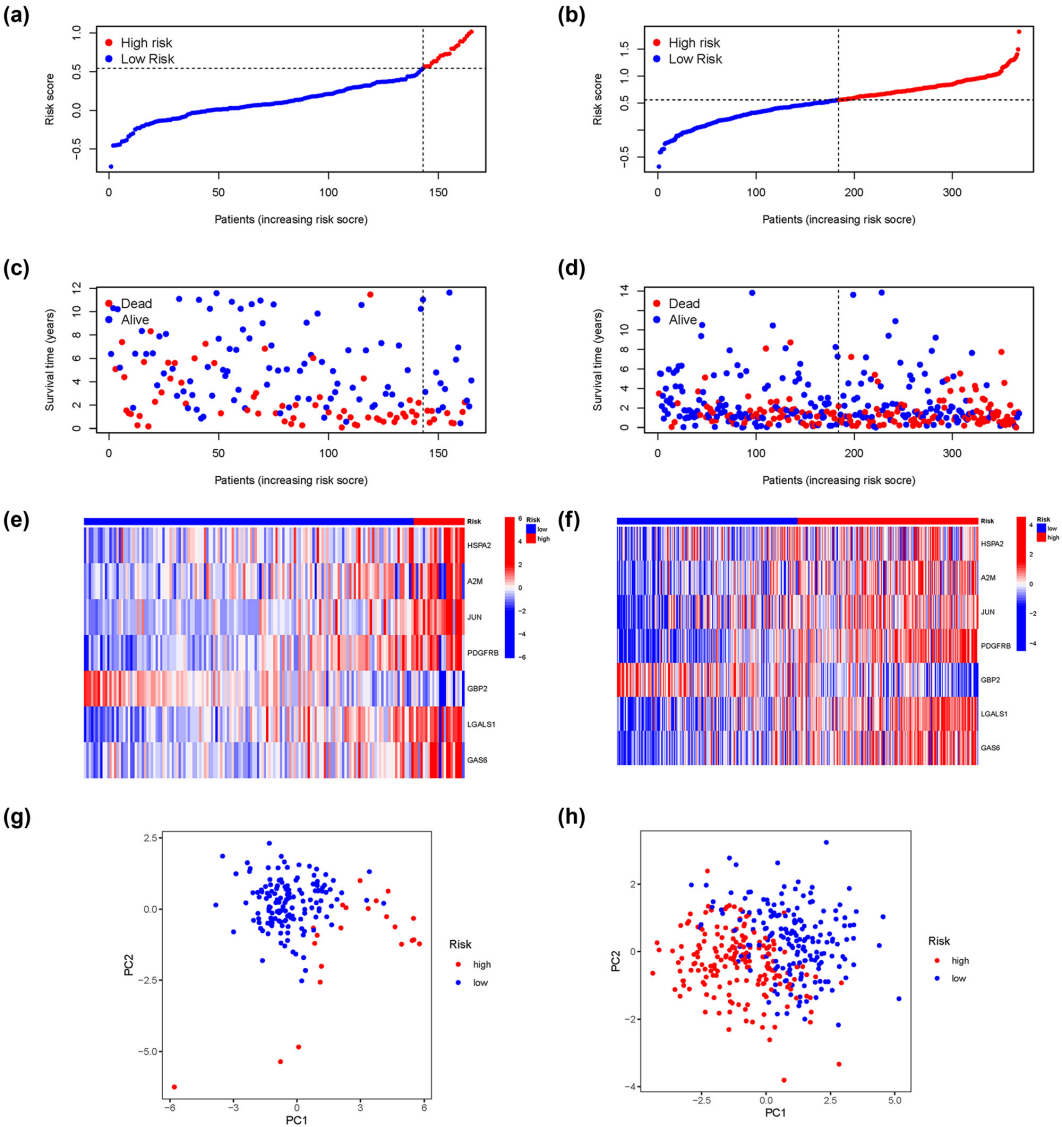
Evaluation of the value of the prognostic model. (a) and (b) Risk score distribution of the patients in TCGA and GEO data sets. (c) and (d) Survival status scatter plots showing poorer prognosis of the high-risk group compared with the low-risk group in two data sets. (e) and (f) Multifactorial heat map showing certain clinical features with a significant correlation with the risk score in two data sets. (g) and (h) PCA analysis in two data sets.

Subsequently, the BLCA patients in the TCGA and GEO datasets were then divided into the low-risk group and high-risk group according to the median risk score in the two data sets (Figure 6a and b). The distribution of survival status between two risk groups in two data sets is shown in Figure 6c and d. As shown in Figure 6e and f by clinical clustering heat-map, we found that the risk score was significantly correlated to TNM stage and grade. Meanwhile, we also found that BLCA patients could be well divided into two categories in both data sets (Figure 6g and h).

### 3.5 Analysis of immune infiltration levels between two risk groups

**Figure 7:**
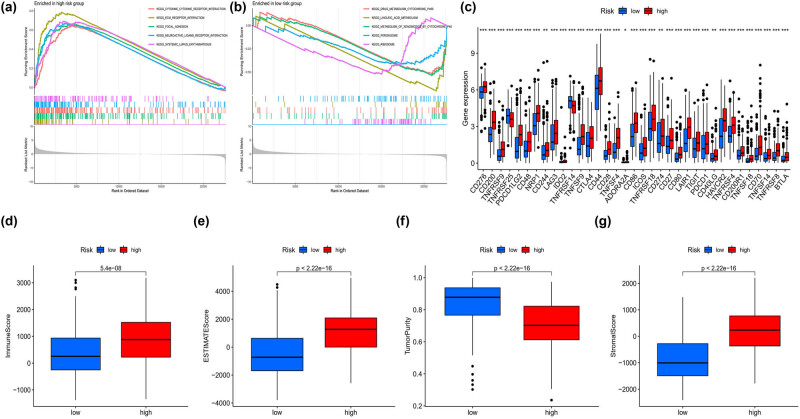
Analysis of immune infiltration and immune checkpoint. (a) and (b) The GSEA enrichment analyses of function pathways between two risk groups in two data sets. (c) The expression of the immune checkpoint genes between two risk groups. (d) Boxplot showing the immune score between two risk groups. (e) Boxplot showing the estimated scores between two risk groups. (f) Boxplot showing the tumor purity scores between two risk groups. (g) Boxplot showing the stromal score between two risk groups.

Through the functional analysis of GSEA, we found that most of the high-risk groups were enriched in immune related functional pathways both in TCGA and in GEO data sets (Figure 7a and b). Meanwhile, most of the immune checkpoints in the high-risk group genes were upregulated (Figure 7c), and the immune score was also higher in the high-risk group (Figure 7d), as well as the estimated score (Figure 7e). Patients in the high-risk group had lower tumor purity scores than those in the low-risk group (Figure 7f). The stromal score was higher in the high-risk group (Figure 7g).

### 3.6 Evaluation of gene mutation between two risk groups

**Figure 8:**
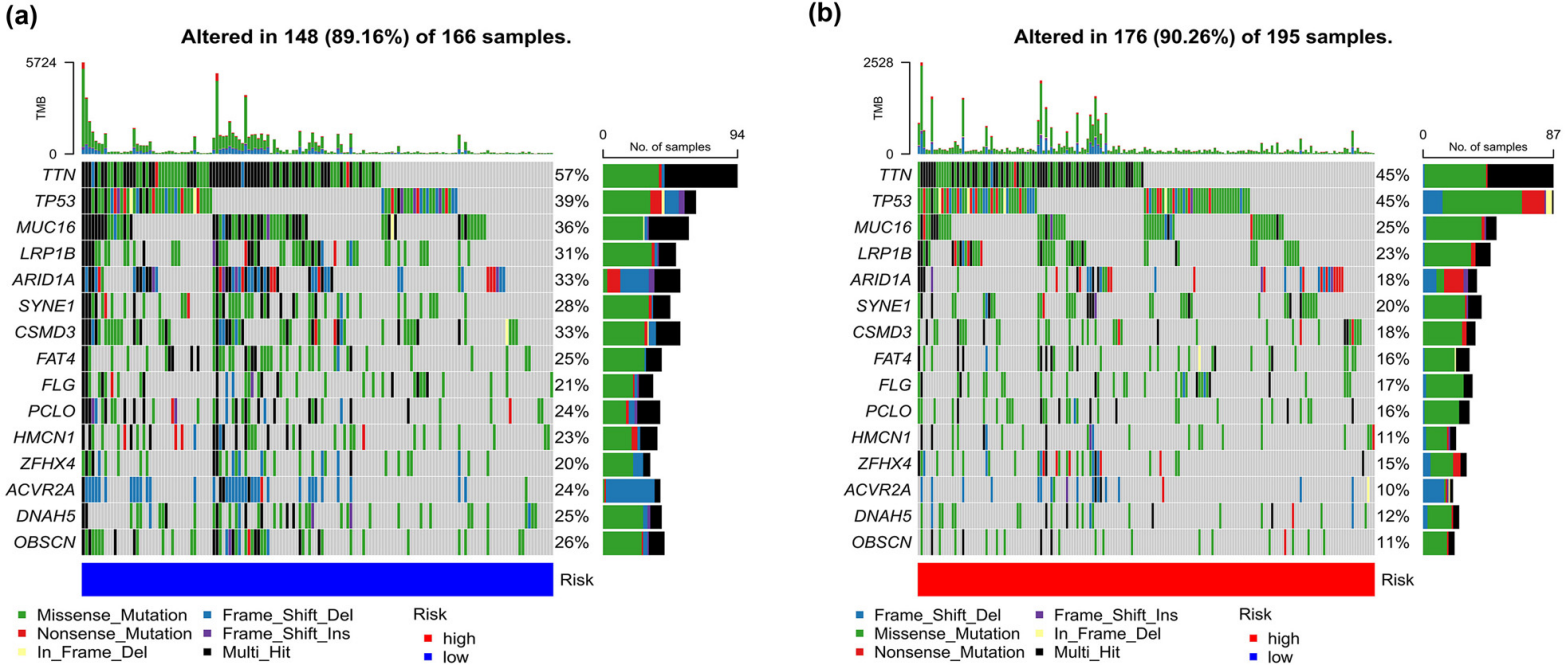
Evaluation of gene mutations between two risk groups: (a) and (b) Heatmaps showing the mutation analysis between high- and low-risk groups in both TCGA and GEO data sets.

Finally, we also analyzed the probability of gene mutation between two risk groups and found 89.16% mutation probability in the high-risk group (Figure 8a) and 90.26% in the low-risk group (Figure 8b). This result suggests that BLCA patients in the low-risk group had more frequent mutations, but their mutations were mostly favorable and led to a better prognosis. In addition, TTN was the most mutated gene, and the missense mutation was the main mutation type between the two risk groups. Our analysis results also revealed that the mutation types of *ARID1A* and *ACVR2A* were mainly nonsense mutation and frame mutation in the high-risk group.

## 4 Discussion

In the past decades, although several advanced methods involved in the diagnosis including molecular biology and imaging, and therapeutic methods such as surgery, chemotherapy, and radiotherapy have been developed and applied [15,16], the therapeutic effect and the 5-year survival of BLCA patients are still poor. Hence, the development of new approaches to diagnosis and treatment involved in subsets and biomarkers is urgent. On the other hand, the poor prognosis of BLCA patients and low prediction efficiency are also some of the high mortalities. Therefore, the investigation of the prognosis model could guide clinical prognostic treatment.

In this study, through analyzing the single cell sequencing data, we obtained seven modeling genes (*HSPA2*, *A2M*, *JUN*, *PDGFRB*, *GBP2*, *LGALS1*, and *GAS6*), which were finally used for constructing a prognosis model. Despite this study, the diagnostic and prognostic values of these relevant genes in BLCA and also other human cancers have been partially studied. *HSPA2*, one DNA methylation biomarker, was identified to be a reliable, noninvasive, and cost-effective diagnostic tool in bladder carcinoma [17]. Lee et al. found that downregulated *ADAMTS1* incorporating *A2M* could contribute to the lung adenocarcinoma progression and change TME, and was also associated with the prognosis of lung adenocarcinoma patients [18]. *JUN* is an inflammation-related gene and participates in the development of different human cancers such as breast cancer [19], non-small cell lung cancer [20], colorectal cancer [21], and recent studies revealed that *JUN* was also significantly correlated to overall survival of cancer patients. Liu et al. found that *PDGFRB* played a crucial role in angiogenesis and tumor cell proliferation development, and this gene was considered as a potential prognostic marker in gastric cancer [22]. *GBP2* was also identified as a prognosis-related biomarker and immunotherapeutic target in several human cancers including colorectal cancer and breast cancer [23,24]. A previous study demonstrated that an increased mRNA level of *LGALS1* was linked to BLCA cell invasiveness and also significantly associated with BLCA prognosis [25]. A recent study revealed that inhibition of GAS6-AS1 axis could efficiently prevent cell propagation and disease development of acute myeloid leukemia [26], suggesting that *GAS6* might be a potential diagnostic and therapeutic target. Although the prognostic values of the several key genes in BLCA have been explored, the significance of a whole based on seven genes joined together in BLCA prognosis remains unclear. In this study, we revealed that these seven genes were most significantly correlated to BLCA overall survival, and we finally constructed the prognostic model using these seven genes and meanwhile confirmed that our prognostic model could accurately predict the patient prognosis of BLCA patients. Our model provided insight into personalized prognostic treatment.

Immune infiltration by T cells admittedly and profoundly influences cancer progression and also in response to immunotherapy [27]. To investigate the impact of immune infiltration on patient prognosis, we analyzed the immune infiltration levels between high- and low-risk groups, and found that most of the high-risk groups were enriched in immune-related functional pathways, and most of the immune checkpoint genes were upregulated in the high-risk group. It suggested that high immune infiltration levels might lead to a better prognosis. In addition, we also analyzed the gene mutations between two risk groups and revealed that patients in the low-risk group have more frequent mutations, suggesting that higher frequent mutations might contribute to a better prognosis. Our findings in the level of immune might account for the diverse prognosis in BLCA patients.

In conclusion, our study constructed a prognostic model using the seven T-cell-related key genes and confirmed that this model could efficiently predict the prognosis of BLCA patients. Meanwhile, we also suggested that diverse levels of immune infiltration and gene mutation probability might be the reason for different prognoses in BLCA. Our results provided novel insight for immunotherapy in BLCA clinically.

## 5 Limitations of study

In order to predict the prognosis of the BLCA, we constructed a prognostic model using the seven T-cell-related key genes. Although this model has been verified *in silico*, future efforts might be needed to directly observe these genes in patient samples.
